# PD-L1 stimulation can promote proliferation and survival of leukemic cells by influencing glucose and fatty acid metabolism in acute myeloid leukemia

**DOI:** 10.1186/s12885-023-10947-7

**Published:** 2023-05-16

**Authors:** Mojdeh Soltani, Mustafa Ghanadian, Behrooz Ghezelbash, Abolfazl Shokouhi, Andrey A. Zamyatnin, Alexandr V. Bazhin, Mazdak Ganjalikhani-Hakemi

**Affiliations:** 1 Department of Immunology, Faculty of Medicine, Isfahan University of Medical Sciences, Isfahan, Iran; 2 Department of Pharmacognosy, School of Pharmacy, Isfahan University of Medical Sciences, Isfahan, Iran; 3 Endocrine and Metabolism Research Center, Isfahan University of Medical Sciences, Isfahan, Iran; 4 Institute of Molecular Medicine, Sechenov First Moscow State Medical University, Moscow, Russia; 5 Department of Biotechnology, Sirius University of Science and Technology, Sochi, Russia; 6 Belozersky Institute of Physico-Chemical Biology, Lomonosov Moscow State University, Moscow, Russia; 7 Department of Immunology Faculty of Health and Medical Sciences, University of Surrey, Guildford, Surrey UK; 8 Department of General, Visceral and Transplant Surgery, Ludwig Maximilians University of Munich, Munich, Germany; 9 Department of Immunology, Faculty of Medicine, Yeditepe University, Istanbul, Turkey

**Keywords:** Acute myeloid leukemia, AML, Fatty acid oxidation, Immunometabolism, Pentose phosphate pathway, Programmed death ligand-1, PD-1

## Abstract

**Background:**

Leukemic cell metabolism plays significant roles in their proliferation and survival. These metabolic adaptations are under regulation by different factors. Programmed Death Ligand -1 (CD-274) is one of the immune checkpoint ligands that do not only cause the immune escape of cancer cells, but also have some intracellular effects in these cells. PD-L1 is overexpressed on leukemic stem cells and relates with poor prognosis of AML. In this study, we investigated effects of PD-L1 stimulation on critical metabolic pathways of glucose and fatty acid metabolisms that have important roles in proliferation and survival of leukemic cells.

**Methods:**

After confirmation of PD-L1 expression by flow cytometry assay, we used recombinant protein PD-1 for stimulation of the PD-L1 on two AML cell lines, HL-60 and THP-1. Then we examined the effect of PD-L1 stimulation on glucose and fatty acid metabolism in cells at the genomic and metabolomic levels in a time dependent manner. We investigated expression changes of rate limiting enzymes of theses metabolic pathways (G6PD, HK-2, CPT1A, ATGL1 and ACC1) by qRT-PCR and also the relative abundance changes of free fatty acids of medium by GC.

**Results:**

We identified a correlation between PD-L1 stimulation and both fatty acid and glucose metabolism. The PD-L1 stimulated cells showed an influence in the pentose phosphate pathway and glycolysis by increasing expression of G6PD and HK-2 (*P* value = 0.0001). Furthermore, PD-L1 promoted fatty acid β-oxidation by increasing expression of CPT1A (*P* value = 0.0001), however, their fatty acid synthesis was decreased by reduction of ACC1 expression (*P* value = 0.0001).

**Conclusion:**

We found that PD-L1 can promote proliferation and survival of AML stem cells probably through some metabolic changes in leukemic cells. Pentose phosphate pathway that has a critical role in cell proliferation and fatty acids β-oxidation that promote cell survival, both are increased by PD-L1 stimulation on AML cells.

## Background

Acute myeloid leukemia (AML) is known as one of the most common and life-threatening groups of leukemias that is caused by genetic abnormalities in hematopoietic stem or progenitor cells and eventually leads to abnormal proliferation and accumulation of undifferentiated and non-functional cells in the bone marrow. It is considered an aggressive disease that requires urgent accurate diagnosis and also timely and effective therapeutic approache. In spite of available remedies which chiefly consist of cytotoxic chemotherapies, the prognosis, especially for older patients remains poor due to tumor regrowth initiated by chemo-resistant leukemic clones after chemotherapy. Hence, more specific and safe therapeutic approaches are needed. According to previous studies, AML cells provide metabolic reprogramming to support their higher survival and elevated rate of growth and proliferation [1]. Enhanced glucose-related metabolic pathways like glycolysis and pentose-phosphate pathway (PPP) and overexpression of some related key enzymes such as pyruvate kinase M2 (PKM2), lactate dehydrogenase A (LDHA) and glucose-6-phosphate dehydrogenase (G6PD), has been observed in AML cell lines and in human primary AML blasts that correlate with drug resistance and poorer prognosis [2]. Furthermore, reprogramming of lipid metabolism has been shown in different myeloid leukemias including AML, and promotes leukemic cell survival and proliferation. Overexpressed carnitine palmitoyltransferase 1A (CPT1A) and carnitine transporter CT2 (SLC22A16) are rate-limiting players of fatty acid oxidation (FAO) has been reported in AML [3]. According to numerous studies, fatty acid metabolism and FAO have an important role in AML survival in the adipocyte-rich BM microenvironment [4].

On the other hand, leukemic cells also overexpress some inhibitory checkpoints or their ligands and therefore, escape from or suppress anti-leukemia immune responses. One of the most important inhibitory pathways utilized by AML cells is PD-1/PD-L1 interaction. PD-L1 is overexpressed on AML cells and is not only considered the main ligand of PD-1 for suppression of immune cells activities but also has some effects on the leukemic cells. This molecule is a type I transmembrane protein and a member of the B7 family encoded by the *Cd274* gene on the human chromosome 9. It has a signal sequence in the intracellular domain that presents three highly conserved sequence motifs (RMLDVEKC, DTSSK, and QFEET) [5]. According to previous studies, PD-L1 has an important role in both the diagnosis and treatment of AML. Its expression on AML cells causes Treg cell expansion and the frequency of PD-1 + Treg cells is considered a potential prognostic predictor in patients with AML [6]. Several studies have confirmed high up-regulation of PD-L1 on immature leukemic precursor cells basically or during treatment, after allogeneic transplantation, and in relapse [7, 8].

Regarding the importance of PD-L1 as the ligand of PD-1 in AML and the various effects of PD-L1 on leukemic cells, we aimed to evaluate its probable influence on some metabolic pathways which are critical in the immortality, proliferation, and survival of AML cells.

## Methods

The ethical aspects of the current study have been approved by Isfahan university of medical sciences ethics committee with ethics code IR.MUI.REC.1400.008.

### Cell culture and treatment

HL-60 and THP-1 cell lines were purchased from Pasteur Institute (Tehran, Iran) and were cultured in Roswell Park Memorial Institute (RPMI) 1640 with 20% heat-inactivated FBS and 1% antibiotics (100 U/ml penicillin and 100 µg/ml streptomycin) (Bio idea, Iran). THP-1 is of monocytic origin and representative of AML M-5 (https://en.wikipedia.org/wiki/THP-1_cell_line) while HL-60 is representative of promyelocytic leukemia M-2 and M-3 (https://en.wikipedia.org/wiki/HL60). According to previous studies, THP-1 and HL-60 cell lines express PD-L1 which can be overexpressed by PMA stimulation [9, 10]. 1 × 10^6^ of each cell line were seeded in each well of the cell culture plate and stimulated with 50 ng/ml phorbol 12-myristate 13 acetate (PMA, Sigma-Aldrich, St Louis, MO, USA) to increase PD-L1 expression. We divided each cell line into two groups including test and control. Both groups were initially stimulated with PMA to express PD-L1. After 24 h of incubation at 37ºC and 5% CO_2_, recombinant human PD-1 (CD279)-Fc chimera (carrier-free) (Biolegend, UK) was added to the medium of test group cells in the concentration of 80 ng/ml while the control group cells were not treated. In order to find the effect of PD-L1 stimulation.

### Flow cytometry analysis

For detection of PD-L1 expression on the cells before their treatment with recombinant PD-1, Flow cytometric analysis was done 24 h after stimulation with PMA. This analysis was accomplished by a FACS Calibur instrument (Becton Dickinson Bioscience, San Jose, USA). Anti-human CD274 (PD-L1)-PE (Biolegend, UK) was applied to measure the expression of PD-L1 protein on HL-60 and THP-1 cells and after that, the results were analyzed using the Cell Quest Pro software (BD Bioscience, USA).

### RNA extraction, cDNA synthesis, and RT-PCR

Total RNA of control and test group cells were extracted after 24, 48 and 72 h following PD-1 treatment using a total RNA extraction kit (Parstus, Inc Iran) according to the manufacturer’s instructions. cDNA synthesis was done immediately after RNA extraction by add Script cDNA synthesis kit (addbio, Korea). The resulting cDNA was applied for real-time quantitative PCR on a Step One Plus™ real-time DNA amplification system (Applied Biosystem, USA) with Ampliqon realQ plus master mix SYBER Green (Denmark) and specific primers. Specific primers for target genes and ACTB, as the housekeeping gene, were designed by Allele ID 7.0 and were ordered to be synthesized by Metabion company (Germany). The primers’ sequences are listed in Table 1.

**Table 1:** The sequences of specific primers that were used in the study

*Genes*	Forward primer sequence	Reverse primer sequence
*β****-actin***	TTCGAGCAAGAGATGGCCA	CACAGGACTCCATGCCCAG
***G6PD***	ATCAGTCGGATACACACATATTC	CGGAACAGCCACCAGATG
***CPT1A***	ACTCACATTCAGGCAGCAAG	ATGGTGTCTGTCTCCTCTCC
***ATGL1***	GCCCAAGCGGAGGATTAC	CAGCAAGCGGATGGTGAAG
***ACC1***	TGAAGCCAAGATAATCCAGCAG	CAAGCCATCCACAATGTAAGC
***HK-2***	AGCCTGGACGAGAGCATC	TCACCACAGCAACCACATC

### Metabolic analysis

#### Gas chromatography/mass spectrometry

Culture supernatants were harvested at the indicated time points (24, 48, and 72 hours after PD-1 treatment) and gas chromatography (GC) analysis was done using Agilent 7890A GC coupled with an Agilent 5975C mass detector with triple quadrupole mass analyzer and electronic ionization (EI) (Agilent Technology, USA). The gas chromatograph was prepared with an HP‐5 GC capillary column (30 m × 0.25 mm; film thickness 0.25 μm) and the oven temperature was started from 100 ºC, held for 2 min, raised by 12ºC/min up to 210°C, followed by 210–250°C by 3ºC/min with the total run time of 34 min. The carrier gas was helium at a flow rate of 2 ml/min and the MSD ChemStation was used as operating software. Before injection the samples into the device, were washed three times with hexane as a nonpolar detergent in order to extract fatty acids. Afterward, they were mixed with adequate amounts of sodium sulfate to eliminate retained water from samples. After GC was carried out, the samples were analyzed on a Thermo-Finnigan Trace DSQ fast-scanning single-quadrupole mass spectrometer.

#### GSH analysis

In order to analyze NADPH production by the pentose phosphate pathway, the oxidation status of cells was examined by measurement of reduced glutathione (GSH). The HL-60 and THP-1cells were grown under the indicated culture conditions at 4×10^6^ cells per 6ml per well in six-well culture plates.72 hours after PD-1 treatment, the cell groups of control and test were harvested and reduced glutathione (GSH) was measured using GSH kit (Navand Salamat, Iran) according to the manufacturer’s instructions.

### Viability analysis (MTT)

For assessing the viability of HL-60 and THP-1 cells before and after treatment with recombinant PD-1, MTT (Methylthiazole tetrazolium) assay was applied. Briefly: 10 µl of a 5 mg/ml MTT (DNA biotech) solution in PBS buffer was added to each well of the 96-well plate and 5 × 10^4^ cells were seeded in each well. After 4-h incubation at 37ºC and 5% CO2, detergent solution (DMSO) was added (100 µl) and Optical density (OD) was measured by a microplate reader (M680 Bio rad, USA) at 570 nm (reference wavelength 690 nm), and the viability of the cells was defined as a percentage compared with untreated (negative) control cells.

### Statistical analysis

Statistical analysis was performed using SPSS 26.0 and Graph Pad Prism 9.1.1. The Kolmogorov–Smirnov test was done to assess the assumption of normality. According to that, the parametric methods were used. The two-way ANOVA and t-test were performed to compare the significance of the difference between two or more groups. *P* < 0.05 was considered to indicate a statistically significant result. Experiment data were presented as mean ± SD. All experiments were performed in triplicates (repeated three times).

## Results

### PMA stimulated Increase of PD-L1 Expression on HL-60 and THP-1 Cells by

**Fig. 1:**
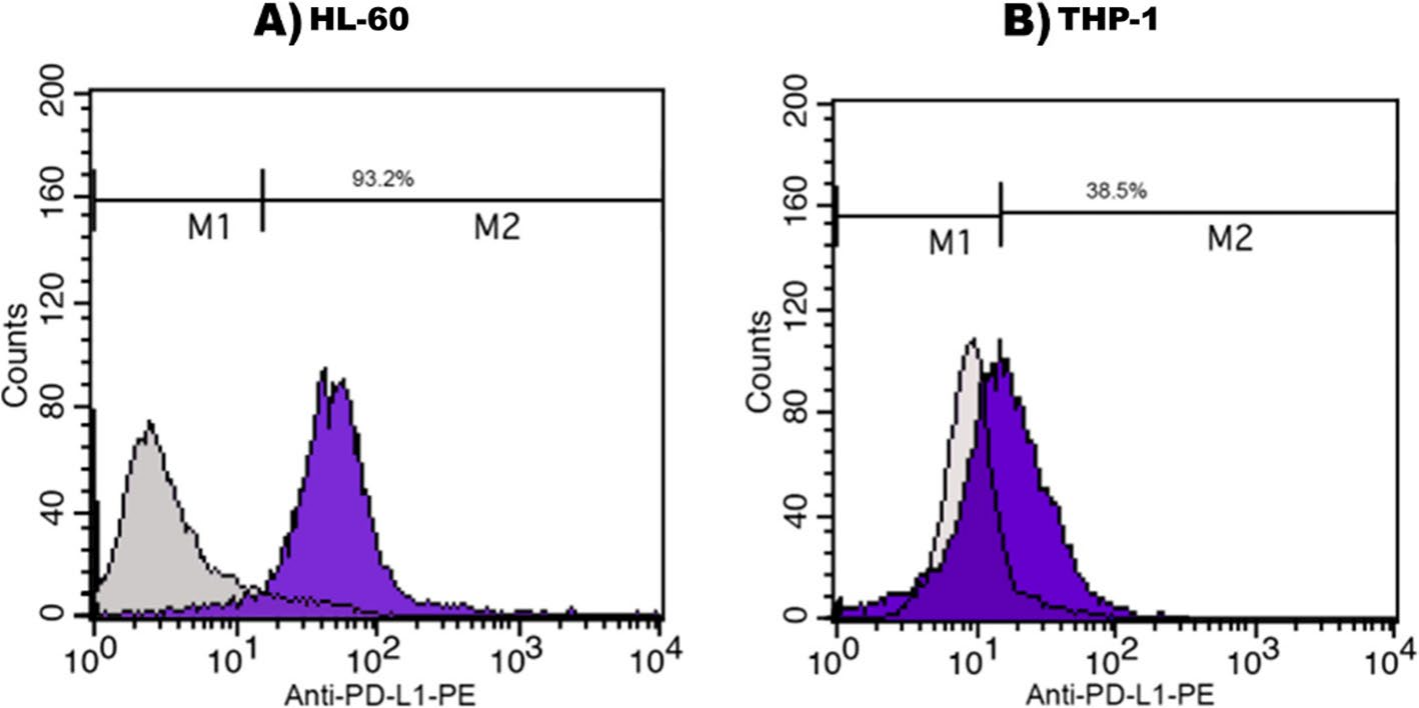
Increasing of PD-L1 expression on HL-60 and THP-1 cells after stimulation with PMA: **A** Histogram graphs of HL-60 cells before and treatment with PMA. PD-L1 expression have obtained to 93.2% after PMA stimulation. **B** Histogram graphs of THP-1 cells before and treatment with PMA. PD-L1 expression have obtained to 38.5% after PMA stimulation

The PD-L1 expression on HL-60 and THP-1 cells was induced with 50 ng PMA. Its expression on the HL-60 cell line was increased from 6.53% up to 93.2% after stimulation for 24 h with PMA, however, it was increased from 5.79% up to 38.5% on THP-1 cells. (Fig. 1).

### PD-1/PD-L1 interaction increased glycolysis and pentose phosphate pathway in HL-60 and THP-1 cell lines

**Fig. 2:**
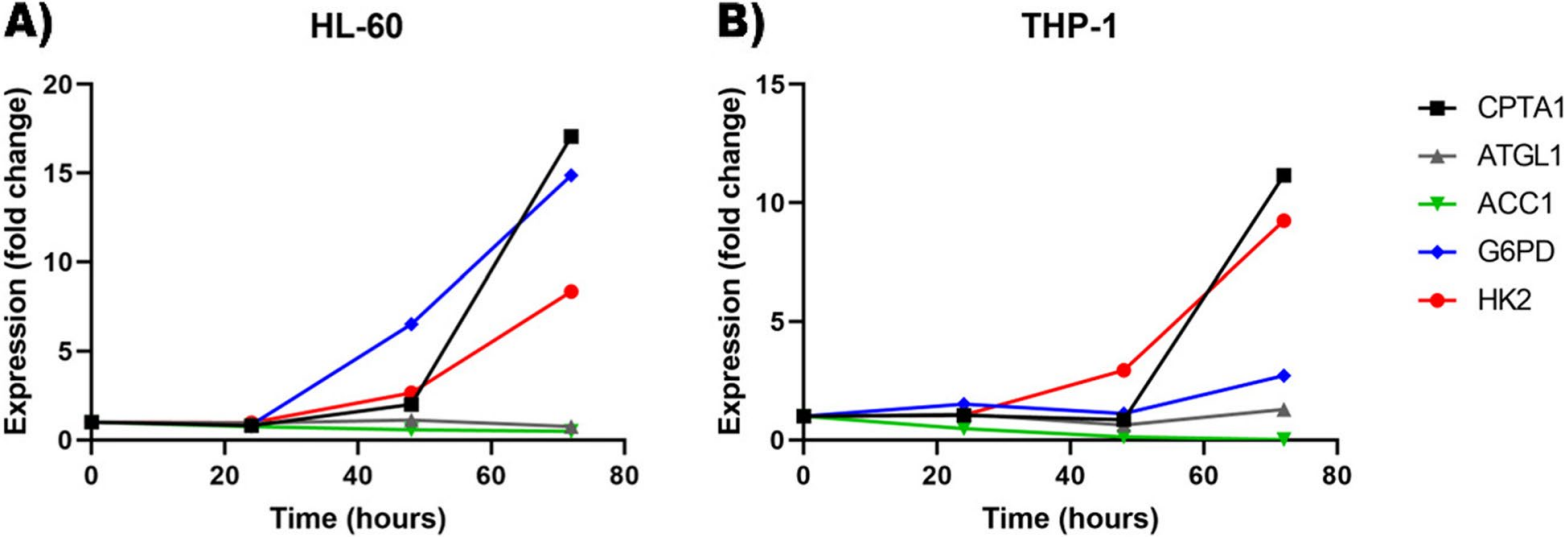
The changes in important enzymes in glucose and fatty acid metabolism after PD-L1 stimulation. **A** enzymes expression changes in HL-60 cells. The graph shows that the expression of CPTA1, G6PD and HK-2 are increasing while ACC1 expression is decreasing significantly due to PD-L1 stimulation. The maximum expression changes in all cases are seen 72 h after stimulation (P Value < 0.05). The ATGL1 didn’t change significantly. (N:3 independent repeats for each experiment). **B** enzymes expression changes in HL-60 cells. The graph shows that the expression of CPTA1, G6PD and HK-2 are increasing while ACC1 expression is decreasing significantly due to PD-L1 stimulation. The maximum expression changes in all cases are seen 72 h after stimulation (P Value < 0.05). The ATGL1 didn’t change significantly. (N:3 independent repeats for each experiment)

To examine the effects of PD-L1 on glucose and fatty acid metabolism, we first divided cells into untreated control and test groups stimulated with recombinant PD-1. The analysis of qRT-PCR results demonstrated that the mean expression of hexokinase (HK-2) was increased in a time-dependent manner. Hexokinase (HK-2) mean expression was followed in 24, 48, and 72 h in control and PD-L1 stimulated cells and the most significant increase was in 72 h. (After *72 h* for HL-60: 8.36 ± 0.2 and for THP-1: 9.24 ± 0.5; vs Control which was 1 ± 0.05; *p* = 0.0001) (Fig. 2) (Table 2).

**Table2:** Mean expression level of enzymes in three time points after PD-L1 stimulation

Enzymes	HL-60 cells				THP-1 cells			
	***control***	***Mean expression in 24 h* ± *SEM***	***Mean expression in 48 h* ± *SEM***	***Mean expression in 72 h* ± *SEM***	***control***	***Mean expression in 24 h* ± *SEM***	***Mean expression in 48 h* ± *SEM***	***Mean expression in 72 h* ± *SEM***
**HK-2**	1 ± 0.05	0.97 ± 0.02 *P* value = 0.9	2.66 ± 0.05 *P* value = 0.0001	8.36 ± 0.2 *P* value = 0.0001	1 ± 0.05	1.05 ± 0.08 *P* value = 0.9	2.94 ± 0.1 *P* value = 0.006	9.24 ± 0.5 *P* value = 0.0001
**G6PD**	1 ± 0.05	0.86 ± 0.04 *P* value = 0.98	6.52 ± 0.06 *P* value = 0.0001	14.88 ± 0.5 *P* value = 0.0001	1 ± 0.05	1.52 ± 0.04 *P* value = 0.01	1.12 ± 0.05 *P* value = 0.7	2.72 ± 0.1 *P* value = 0.0001
**CPT1A**	1 ± 0.05	0.81 ± 0.04 *P* value = 0.8	2.01 ± 0.05 *P* value = 0.01	17.05 ± 0.3 *P* value = 0.0001	1 ± 0.05	1.04 ± 0.04 *P* value = 0.1	0.86 ± 0.04 *P* value = 0.9	11.16 ± 0.5 *P* value = 0.0001
**ACC**	1 ± 0.05	0.75 ± 0.03 *P* value = 0.007	0.58 ± 0.02 *P* value = 0.0001	0.49 ± 0.02 *P* value = 0.0001	1 ± 0.05	0.48 ± 0.06 *P* value = 0.001	0.13 ± 0.06 *P* value = 0.0001	0.02 ± 0.002 *P* value = 0.0001
**ATGL**	1 ± 0.05	0.95 ± 0.04 *P* value = 0.8	1.13 ± 0.04 *P* value = 0.27	0.76 ± 0.02 *P* value = 0.032	1 ± 0.05	1.09 ± 0.08 *P* value = 0.9	0.63 ± 0.05 *P* value = 0.1	1.29 ± 0.1 *P* value = 0.22

Glucose-6-phosphate dehydrogenase (G6PD) expression was also increased during three-time points and the most significant increase was observed in 72 h. (After *72 h* for HL-60: 14.88 ± 0.5 and for THP-1: 2.72 ± 0.1 vs Control which was 1 ± 0.05; *p* = 0.0001) (Fig. 2) (Table 2).

### PD-1/PD-L1 interaction increased fatty acids oxidation and decreased fatty acid synthesis in HL-60 and THP-1 cell lines

Carnitine palmitoyl transferase (CPT1A) expression increased in a time dependent manner in both cell lines stimulated with PD-1 compared with control cells and the most significant increase was in 72 h. (*24 h* mean in HL-60 and THP-1: 0.81 and 1.04 ± 0.04 ؛*48 h* mean in HL-60 and THP-1: 2.01 ± 0.05 and 0.86 ± 0.04 ؛*72 h* mean in HL-60 and THP-1: 17.05 ± 0.3 and 11.16 ± 0.5 vs Control mean = 1 ± 0.05) (Table 2). Despite CPTA1, the expression of adipose triglyceride lipase (ATGL1) didn’t show a significant change in PD-L1 stimulated compared with control groups. (*24 h* mean in HL-60 and THP-1: 0.95 ± 0.04 and 1.09 ± 0.08 ؛*48 h* mean in HL-60 and THP-1: 1.13 ± 0.04 and 0.63 ± 0.05 ؛*72 h* mean in HL-60 and THP-1: 0.76 ± 0.02 and 1.29 ± 0.1 vs Control mean = 1 ± 0.05) (Fig. 2) (Table 2).

Although fatty acid oxidation increased after PD-L1 stimulation, the analysis of the expression of acetyl-coA carboxylase 1 (ACC1) demonstrated that it was decreased in PD-L1 stimulated cell groups compared with control. ACC1 expression changes were followed in 24, 48, and 72 h after PD-L1 stimulation and the most significant reduction was in 72 h. (*24 h* mean in HL-60 and THP-1: 0.75 ± 0.03 and 0.48 ± 0.06 ؛*48 h* mean in HL-60 and THP-1: 0.58 ± 0.02 and 0.13 ± 0.06 ؛*72 h* mean in HL-60 and THP-1: 0.49 ± 0.02 and 0.02 ± 0.002 vs Control mean = 1 ± 0.05) (Fig. 2) (Table 2).

### PD-L1 stimulation influenced free fatty acids levels in the cell culture supernatant

**Fig. 3:**
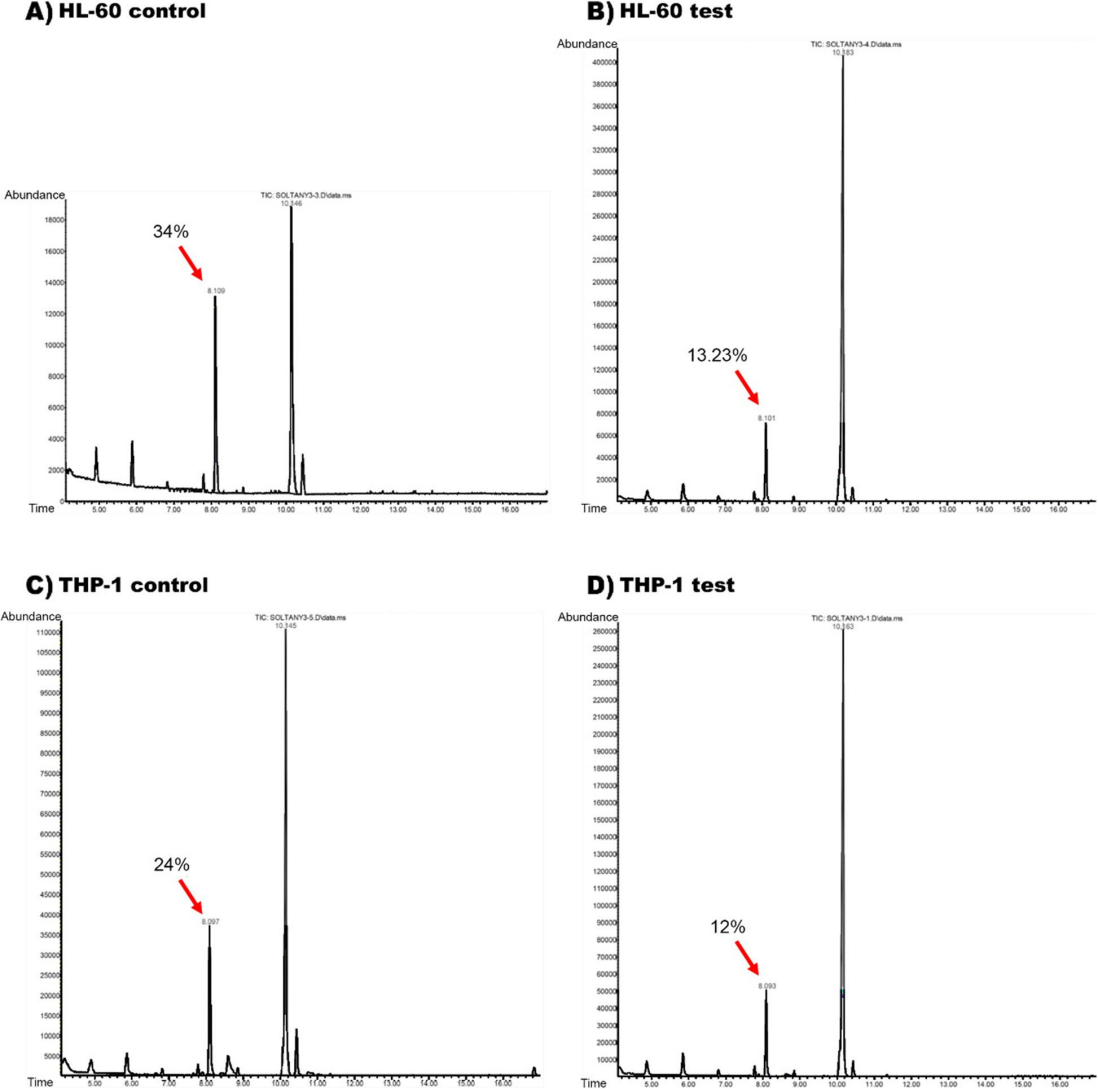
The free fatty acids gas chromatography analysis in supernatant of PD-L1 stimulated cells compared with control cells. **A** The relative abundance of free fatty acids in the control HL-60 media. Determined peak shows palmitate which composes 34% of total detected fatty acids in control HL-60 cells supernatant (without PD-L1 stimulation). **B** The relative abundance of free fatty acids in HL-60 supernatant 72 h after PD-L1 stimulation. Determined peak shows palmitate relative abundance decreased to 13.23%. **C** The relative abundance of free fatty acids in the control THP-1 media. Determined peak shows palmitate composes 24% of total detected fatty acids in control THP-1 cells (without PD-L1 stimulation). Determined peak shows palmitate which composes 24% of total detected fatty acids. **D** The relative abundance of free fatty acids in THP-1 supernatant 72 h after PD-L1 stimulation. Determined peak shows palmitate relative abundance decreased to 12%

In order to examine the production or consumption of fatty acids by cells, we analyzed and compared the relative abundance changes of free fatty acids in both the test and control medium 72 h after PD-1 treatment. We followed the palmitic acid relative abundance changes as the main substrate for fatty acid β-oxidation. According to gas chromatography analysis, the initial relative abundance of palmitic acid in the medium was 34% and 24% in untreated cells supernatant and it decreased in medium of the test group 72 h after treatment. It declined to 13.23% and 12% in the supernatant of HL-60 and THP-1 cells, respectively (Fig. 3).

### Reduced glutathione (GSH) increased after PD-L1 stimulation

**Fig. 4:**
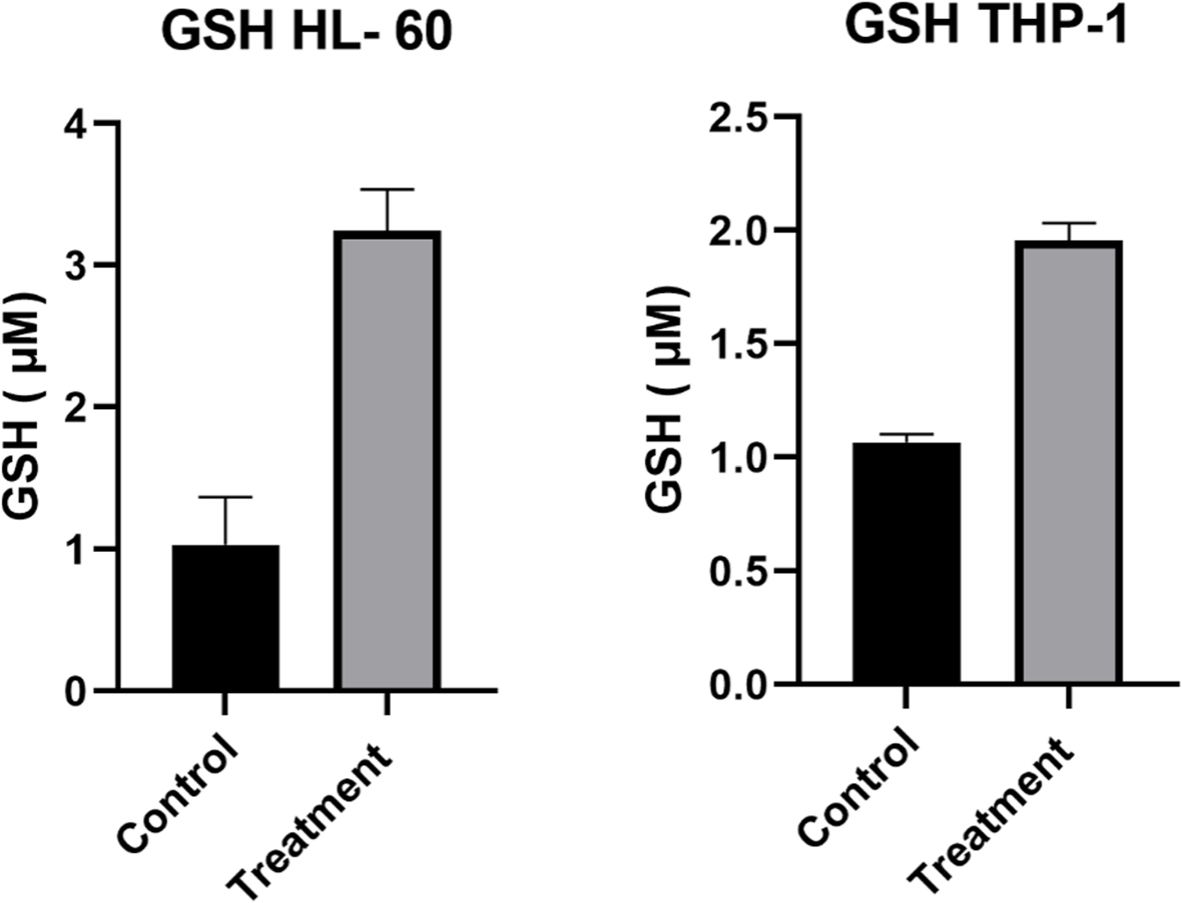
Comparison of reduced glutathione (GSH) between PD-L1 stimulated and control Cells. The amount of GSH was measured 72 h after PD-L1 stimulation when we had fined the most enzyme expression changes. The reduced glutathione was more in PD-L1 stimulated compared with control cells in both HL-60 and THP-1 cells (Unpaired t test, P value = 0.004 and 0.019 respectively)

NADPH, as the important cofactor of glutathione reductase, is mainly produced in the pentose phosphate pathway. Thus, we analyzed the reduced form of glutathione which is an indicator of NADPH production and the status of oxidation reactions. We compared GSH content in HL-60 and THP-1 cells in both the control and test groups after 72 h of stimulation. The GSH amount was increased in cells after PD-1 treatment. In HL-60 cells the mean concentration was 3.24 µM in PD-L1 stimulated vs 1.03 µM in the control group, and in THP-1 cells the mean concentration was 1.06 µM vs 1.95 µM. (*P* Value = 0.004 and 0.01 for HL-60 and THP-1 cells respectively) (Fig. 4).

### Stimulation of PD-L1 increased cell viability

**Fig. 5:**
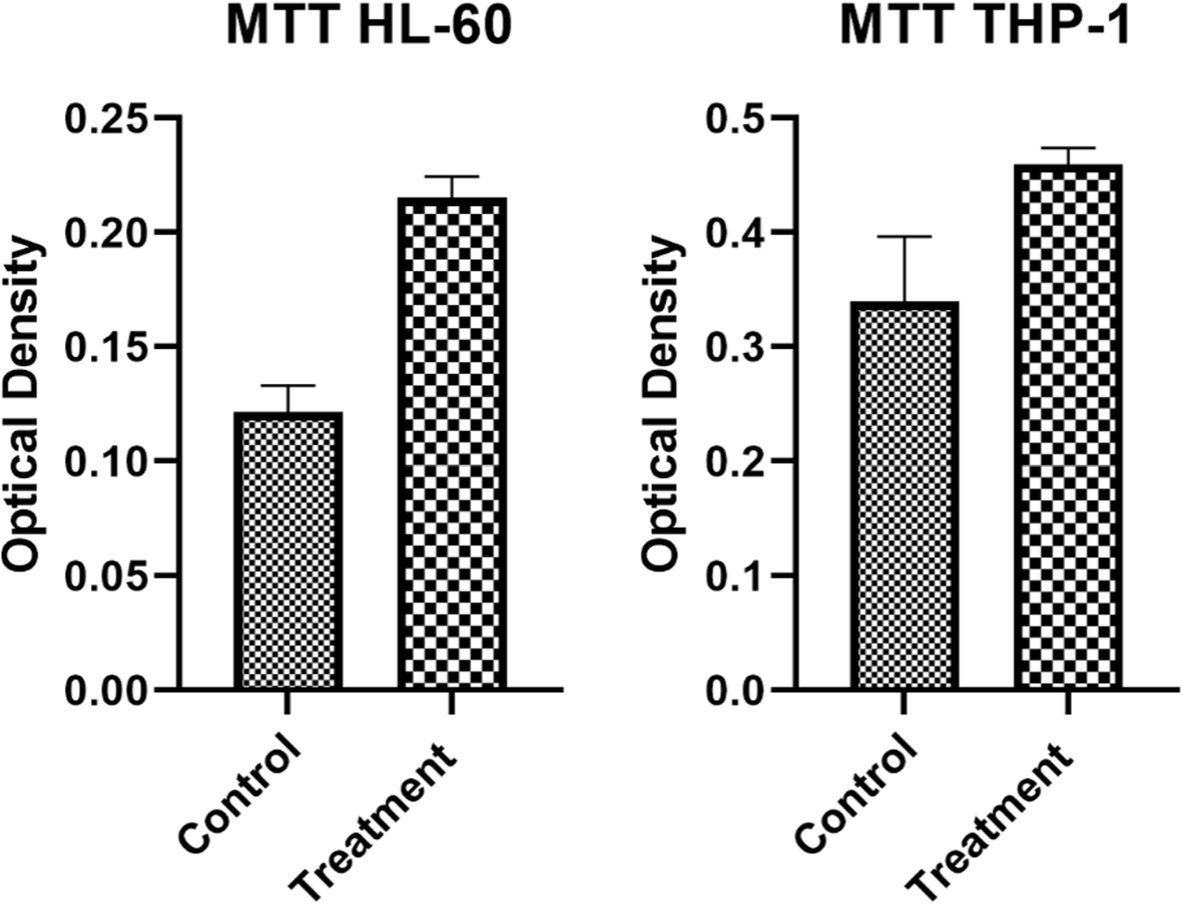
Comparison of cells viability between PD-L1 stimulated and control Cells. Cell viability was measured by MTT test 72 h after PD-L1 stimulation when we had fined the most enzyme expression changes. Cell viability was more in PD-L1 stimulated compared with control cells in both HL-60 and THP-1 cells (Unpaired t test, value = 0.0001 in both)

To assess the effects of PD-L1 stimulation on THP-1 and HL60 cell viability, the MTT assay was applied 72 h after the treatment of cells with recombinant PD-1. The viability of both cell lines was increased in the test group compared to the control group (Fig. 5).

## Discussion

An increasing number of studies have demonstrated the importance of cell metabolism in AML occurrence, development, and drug resistance. Various factors including tumor microenvironment and some cell-intrinsic signaling pathways are involved in the metabolic adaptation of leukemic cells. Furthermore, inhibitory checkpoints and their ligands can be effective in this era, particularly regarding their overexpression on the leukemic cells. The present study examined the influence of programmed death ligand 1 (PD-L1) on some metabolic pathways which play critical roles in tumor cells’ survival and proliferation.

Following previous studies, we examined the PD-L1 stimulation on glucose and fatty acid metabolism which are critical metabolic pathways for the proliferation and survival of leukemic cells. We found that PD-L1 stimulation in both HL-60 and THP-1 AML cell lines alters the glucose and fatty acids metabolism and their proliferation. Although our results were more significant in the HL-60 cell line, it may be due to more PD-L1 expression in these cells compared with THP-1. We found that PD-L1 increases glycolysis and pentose phosphate pathway (PPP) by overexpression of HK-2 and G6PD as the key regulators of these pathways. Moreover, we showed that PD-L1 promotes GSH amounts in the AML cells which may be related to increased NADPH production in PPP. These metabolic effects can occur because of PI3K/Akt pathway activation downstream of PD-L1. Similar findings have been obtained in acute myeloid leukemia where Ma et al. have reported in AML cell lines that glycolysis‑associated genes ALDOA, HK2, LDHA, and PGK1 were highly expressed on PD‑L1 high‑expressing cell lines [10]. Previous studies on some other malignancies have shown comparable findings. Cui et al. have reported the impact of PD-L1 on the glucose metabolism of lung adenocarcinoma cells. They showed that the upregulation of PD-L1 can affect the expression of glycolysis-related enzymes/proteins, glucose uptake, and lactate production via PI3K/Akt pathway [11]. PD-L1 knockdown in a mouse sarcoma model caused the reduction of the extracellular acidification rate (ECAR). Furthermore, it decreased the expression of glycolysis enzymes, showing PD-L1’s role in glucose consumption through the ATK/mTOR pathway in cancer cells [12]. Although PD-L1 increases glucose metabolism in tumor cells, Patsoukis et al. have reported that PD-1 inhibits glycolysis in T cells [13]. So, it is concluded that the PD-1/PD-L1 pathway not only suppresses anti-tumor activities but also reinforces tumor cell proliferation.

Our findings have also shown that PD-L1 stimulation promotes fatty acid oxidation (FAO) while reducing fatty acids synthesis (FAS). We found that it decreases fatty acid synthesis by reduction of ACC1 expression. We also showed that the expression of carnitine palmitoyl transferase (CPT1A) as the key regulator of FAO was increased, however, we didn’t find any significant changes in the expression of adipose triglyceride lipase (ATGL1). ATGL1 hydrolyzes triglyceride by separation of its fatty acids and therefore, it is considered a lipolysis indicator. This shows that PD-L1 may just regulate free fatty acids metabolism. It can also be due to the fact that fatty acid oxidation and synthesis are regulated by the PI3K/Akt/mTOR pathway while lipolysis is not. Accordingly, Xu et al. demonstrated that miR-421 promotes fatty acid oxidation via activating PI3K/AKT/mTOR pathway in non-small cell lung cancer [14]. According to Lin et al. study, PD-L1 was reported to increase cancer lipid uptake by upregulating fatty-acid binding protein (Fabp) 4 and 5 expression in gastric adenocarcinoma [15]. The metabolic effects of PD-1 on T cells were somewhat different. PD-1 promotes FAO of endogenous lipids, and induces lipolysis as indicated by elevation of the lipase ATGL, the lipolysis marker glycerol, and the release of fatty acids. Conversely, CTLA-4 inhibits glycolysis without augmenting FAO [13]. These findings may bold the effects of the microenvironment and its nutrition competition which can effectively influence metabolic changes in both immune and tumor cells. Contrary to most previous studies which genetically manipulated the expression of PD-L1, we stimulated the cell surface PD-L1 with its natural receptor (PD-1) to make the conditions more similar to the natural situation. However, checkpoints stimulation or inhibition while leukemic cells are co-cultured with T cells is recommended for future studies.

Recent studies have identified cancer cell-intrinsic PD-L1 signaling is associated with sustaining cancer survival and proliferation [16]. A growing number of studies have suggested that PD-L1 (CD-274) is involved in cancer-cell metabolic adaptations. On the other hand, the crucial role of metabolic pathways like the pentose phosphate pathway and fatty acid metabolism in the proliferation and survival of malignant tumors has been proven [17–19]. Therefore, it can be concluded that PD-L1 signaling increases cell survival and proliferation, maybe through increasing some metabolic pathways like pentose phosphate and fatty acid oxidation. This metabolic change can be a molecular mechanism for the studies that showed proliferation increase due to PD-1/PD-L1 interaction. For example, Wang et al. reported that PD‑L1 regulates cell proliferation and apoptosis in acute myeloid leukemia by activating PI3K‑AKT signaling pathway [20]. On the other hand, our finding shows that PD-L1 stimulation increased fatty acid oxidation in AML cell lines. Regarding the vital role of this metabolic pathway in cell survival and drug resistance, PD-L1 can promote AML blast cells survival through a metabolic alteration [4, 21, 22].

## Conclusion

In conclusion, our present study showed that PD-1/PD-L1 interaction induces metabolic reprogramming in AML cell lines by stimulation of glycolysis, pentose phosphate pathway, and fatty acids oxidation while declining fatty acid synthesis. These metabolic changes may eventually promote AML cells survival and proliferation, therefore making their components potential therapeutic targets. Our study is a pilot one for designing combinational treatments that target immune checkpoints and metabolic pathways.

## Data Availability

All allowed and requisite datasets generated and/or analyzed during the current study are provided in the manuscript (“Table 2” and “Fig. 3” are raw data that have been presented). More detailed data are not publicly available based on the regulations and restrictions of the study grant supporter. For more information, please contact to “mghakemi@med.mui.ac.ir”.

## Abbreviations

PD-L1: Programmed Death Ligand -1 (CD-274)
AML: Acute myeloid leukemia
PPP: Pentose Phosphate Pathway
FAO: Fatty Acids Oxidation
HK-2: Hexokinase
G6PD: Glucose-6-phosphate dehydrogenase
CPT1A: Carnitine palmitoyl transferase
ATGL1: Adipose Triglyceride Lipase
ACC1: Acetyl-CoA Carboxylase 1
